# Signatures of Thalamocortical Alpha Oscillations and Synchronization With Increased Anesthetic Depths Under Isoflurane

**DOI:** 10.3389/fphar.2022.887981

**Published:** 2022-06-03

**Authors:** Jingyao Jiang, Yi Zhao, Jin Liu, Yaoxin Yang, Peng Liang, Han Huang, Yongkang Wu, Yi Kang, Tao Zhu, Cheng Zhou

**Affiliations:** ^1^ Department of Anesthesiology, West China Hospital of Sichuan University, Chengdu, China; ^2^ Laboratory of Anesthesia and Critical Care Medicine, National-Local Joint Engineering Research Centre of Translational Medicine of Anesthesiology, West China Hospital of Sichuan University, Chengdu, China; ^3^ Department of Anesthesiology, West China Second Hospital of Sichuan University, Chengdu, China; ^4^ Intelligent Manufacturing Institute, Chengdu Jincheng College, Chengdu, China

**Keywords:** computational model, electroencephalogram, phase–amplitude coupling, isoflurane, thalamocortical networks, alpha oscillations

## Abstract

**Background:** Electroencephalography (EEG) recordings under propofol exhibit an increase in slow and alpha oscillation power and dose-dependent phase–amplitude coupling (PAC), which underlie GABA_A_ potentiation and the central role of thalamocortical entrainment. However, the exact EEG signatures elicited by volatile anesthetics and the possible neurophysiological mechanisms remain unclear.

**Methods:** Cortical EEG signals and thalamic local field potential (LFP) were recorded in a mouse model to detect EEG signatures induced by 0.9%, 1.5%, and 2.0% isoflurane. Then, the power of the EEG spectrum, thalamocortical coherence, and slow–alpha phase–amplitude coupling were analyzed. A computational model based on the thalamic network was used to determine the primary neurophysiological mechanisms of alpha spiking of thalamocortical neurons under isoflurane anesthesia.

**Results:** Isoflurane at 0.9% (light anesthesia) increased the power of slow and delta oscillations both in cortical EEG and in thalamic LFP. Isoflurane at 1.5% (surgery anesthesia) increased the power of alpha oscillations both in cortical EEG and in thalamic LFP. Isoflurane at 2% (deep anesthesia) further increased the power of cortical alpha oscillations, while thalamic alpha oscillations were unchanged. Thalamocortical coherence of alpha oscillation only exhibited a significant increase under 1.5% isoflurane. Isoflurane-induced PAC modulation remained unchanged throughout under various concentrations of isoflurane. By adjusting the parameters in the computational model, isoflurane-induced alpha spiking in thalamocortical neurons was simulated, which revealed the potential molecular targets and the thalamic network involved in isoflurane-induced alpha spiking in thalamocortical neurons.

**Conclusion:** The EEG changes in the cortical alpha oscillation, thalamocortical coherence, and slow–alpha PAC may provide neurophysiological signatures for monitoring isoflurane anesthesia at various depths.

## Introduction

The state of general anesthesia is a pharmacologically induced, reversible state mainly characterized by unconsciousness, amnesia, and immobility (Brown et al., 2010). An appreciation of the systemic effects of general anesthetics is necessary to understand how these agents elicit different states of arousal and unconsciousness (Franks, 2008; Brown et al., 2010; Brown et al., 2011).

The understanding of how general anesthetics modulate the central nervous system can provide new insights into monitoring the depth of general anesthesia, which may avoid the side effects of clinical anesthesia, mainly including intraoperative awareness, delayed emergence, postoperative delirium, and cognitive dysfunction (Cascella, 2016; Lobo et al., 2021). The characteristics of electroencephalogram (EEG) are obviously significant for monitoring brain function under general anesthesia. The prominent EEG features in the propofol-induced unconsciousness exhibit an increased power of slow oscillations (Alkire et al., 2008; Purdon et al., 2015), alpha oscillations (Purdon et al., 2013; Akeju et al., 2014), and concentration-dependent PAC (phase–amplitude coupling) between slow and alpha oscillations (Mukamel et al., 2011; Purdon et al., 2013; Mukamel et al., 2014). Many studies have identified the underlying mechanisms of general anesthetic-induced alpha oscillations. For example, studies on neural circuits demonstrate that a decreased neural fluctuation intensity in the ascending arousal system (Hutt et al., 2018), a prolongation of the transmission delay of corticothalamic circuit (Hashemi et al., 2017), and differential neural structures in the corticothalamic circuit (Kratzer et al., 2017) are involved in alpha oscillations under general anesthesia. At the molecular level, previous works suggest the primary importance of γ-aminobutyric acid (GABA) augmentation in propofol-induced alpha oscillations (Alkire et al., 2008; Franks, 2008; Flores et al., 2017). Some mathematical models have simulated the generation of propofol-induced alpha oscillations and generation of PAC between alpha and slow oscillations as a result of potentiation of GABA_A_ synaptic transmission (Ching et al., 2010; Soplata et al., 2017).

However, compared with intravenous general anesthetic propofol, there is a relative paucity of systematic studies on volatile anesthetics. Understanding the EEG signatures at various anesthetic depths under volatile anesthetics is also important for monitoring brain functions under volatile anesthesia (Cascella, 2016; Lobo et al., 2021). Unlike propofol, which has a primary selective action on the γ-aminobutyric acid (GABA_A_) receptor (Hemmings et al., 2005; Franks, 2008; Hemmings et al., 2019), volatile anesthetics act on a diverse set of molecular targets, mainly including the GABA_A_ receptor (Olsen and Li, 2011; Kotani and Akaike, 2013), the N-methyl-D-aspartate (NMDA) receptor (Ming et al., 2001), background potassium channels (K_Leak_) (Li et al., 2018), and the α-amino-3-hydroxy-5-methyl-4-isoxazole-propionic acid (AMPA) receptor (Joo et al., 2001). Thus, an enhanced GABAergic inhibition is likely to be an important but not the only mechanism for volatile anesthetic-induced general anesthesia and EEG changes. Therefore, the changes in EEG under volatile anesthetics may also exhibit differential signatures from those induced by propofol.

In this study, cortical EEG and thalamic LFP were recorded in a mouse model to evaluate the EEG signatures under the classical volatile anesthetic isoflurane. We hypothesized that the EEG changes in the cortical alpha oscillation, thalamocortical coherence, and slow–alpha PAC may provide neurophysiological signatures for monitoring isoflurane anesthesia at various depths.

## Methods

### Animals

All the experimental protocols were approved by the Institutional Animal Experimental Ethics Committee of West China Hospital of Sichuan University (Chengdu, China). Animal Research Reporting *In Vivo* Experiments (ARRIVE) guidelines were applied during the study. Adult C57 BL/6J mice (8 weeks old) were housed in humidity- and temperature-controlled cages, underwent a 12-h light–dark cycle (light on from 7 a.m. to 7 p.m.), and had ad libitum access to chow and water. Animals were euthanized using CO_2_ and isoflurane after experiments.

### Determination of Minimum Alveolar Concentrations

Minimum alveolar concentrations (MACs) of isoflurane that induced loss of righting reflex (LORR) and loss of movements to tail-clamping stimulus (LOM) were measured, respectively, as MAC_LORR_ and MAC_LOM_ (Fukagawa et al., 2014; Zhou et al., 2015). Isoflurane was delivered in an open-circuit rodent anesthetic system. Carrier gas flow was 2 L/min (100% O_2_). For the measurement of LORR, each mouse was placed in a cylindrical anesthetic chamber (15 cm in length and 5 cm in diameter). Isoflurane concentrations were increased starting from 0.6% in a step interval of 0.05%. As the plastic chambers were rotated slowly, LORR was defined as the mouse loss ability to turn itself prone onto all four limbs in 60 s. For the measurement of LOM, mice were placed in a cuboid container (25 cm in length, 15 cm in width, and 12 cm in height) and left the tail outside. Isoflurane concentrations were increased from 0.9% in a step interval of 0.05%. LOM was defined as no purposeful movements to tail-clamping stimulation in 30 s.

### Implantation of Electroencephalogram Electrodes and Intracranial Electrodes

Four hand-made EEG electrodes were implanted in each animal, including two recording electrodes, a common electrode, and a grounding electrode. The cortical EEG electrode, common electrode, and grounding electrode were anchor screws (0.6 mm in diameter and 1.5 mm in length), which were fixed on the skull and inserted into the cortex. The thalamic electrode was made by insulating silver wire (0.2 mm in diameter) and connected with an anchor screw. The thalamic electrode was made by insulating silver wire (0.2 mm in diameter). The insulation was removed from one end of the wire, and the other end was cut with a conductive tip for intracranial insertion (Guidera et al., 2017). When implanting EEG screws and thalamic electrodes, mice were anesthetized with 2% isoflurane and placed in a stereotactic apparatus (RWD, Shenzhen, China). A heating pad (RWD, Shenzhen, China) was used to maintain the body temperature of mice. A longitudinal incision was made to expose the bregma and lambda of the skull. The cortical EEG electrode was implanted to primary motor cortex (M1) [anteroposterior (AP): +1.0 mm; mediolateral (ML): +1.5 mm; and dorsoventral (DV): –1.5 mm] to record the frontal cortex EEG. The thalamic electrode was inserted into ventroposterolateral and/or ventroposteromedial thalamic nuclei (VPL/VPM) [AP: –1.5 mm; ML: +1.5 mm; and DV: –3.5 mm], which was close to relay cells to record thalamic LFPs (Landisman and Connors, 2007; Haidarliu et al., 2008) (Supplementary Figure S1). The common electrode was implanted in the left frontal cortex [AP: +1.0 mm; ML: –1.5 mm; and DV: –1.5 mm]. The grounding electrode was planted in the parietooccipital cortex [AP: –3.5 mm; ML: –1.5 mm; and DV: –1.5 mm] to eliminate the disturbance of the surrounding environment. Four electrodes were connected to a miniature plug with silver wires. Then, the electrodes and the plug were secured to the skull with dental acrylic. After the surgery, animals were allowed to recover for 1 week before recordings.

### Recordings of Frontal Cortex EEG and Thalamic LFPs

Electrophysiological signals (frontal cortical EEG and/or thalamic LFPs) were recorded by a Pinnacle EEG recording system (Part #8200-SL; Pinnacle Technology, United States) (Gelegen et al., 2021). A preamplifier unit was rigidly attached to the miniature plug, providing the first stage of amplification (×100) and initial high-pass filtering (first-order 0.5 Hz for EEG). The recording sampling frequency of signals was 500 Hz. Raw signals were preamplified, digitized, and recorded using a Sirenia Acquisition system (Part #8206-SL; Pinnacle Technology, United States) and analyzed using MATLAB (version 2006a; MathWorks, United States). The accuracy of the electrode placement was confirmed by visual examination of brain tissue in postmortem.

For the recordings under isoflurane, each mouse was placed in a transparent gastight plastic chamber (20 cm in length, 15 cm in width, and 10 cm in height). A heating pad (RWD, Shenzhen, China) was used to maintain the body temperature of mice. The chamber was ventilated with 2 L/min 100% O_2_, and the EEG and LFPs were recorded for 5 min as baseline. Then, the concentrations of isoflurane were continuously increased from 0.9% to 1.5% and then to 2.0% and decreased to 1.5, 0.9, and 0.5%. The concentrations of isoflurane were continuously monitored by an infrared gas monitor (Datex-Ohmeda, WI, United States). Each concentration of isoflurane was maintained for at least 20 min. Frontal cortical EEG and thalamic LFPs were recorded continuously. For the recordings under propofol, propofol (AstraZeneca SpA, London, United Kingdom) (Ou et al., 2017) was intravenously injected into the caudal vein of mice at a dose of 14 mg/kg. After injection, the duration of LORR was recorded. The definition of LORR was the same as described in isoflurane anesthesia. Cortical EEG and thalamic LFPs were continuously monitored throughout.

### Spectral and Coherence Analysis of Cortical EEG and Thalamic Local Field Potentials

Power spectra were estimated using multitaper spectral methods implemented in the Chronux toolbox with the “mtspectrumc” or “spectrograms” function in MATLAB (version 2006a; MathWorks, United States) (Bokil et al., 2007). Spectrograms were computed using the continuously recorded EEG signals from 0.5 to 25 Hz. The spectral difference at differential isoflurane concentrations was calculated for particular EEG epochs. Typical frontal cortical EEG and thalamic LFPs were analyzed at differential isoflurane concentrations for the duration of 2 min. For baseline EEG and/or LFPs, stable signals without artifacts were chosen. For each concentration of isoflurane, the signals were chosen at 10 min after stabilization of isoflurane at this concentration. For propofol, 30-s duration signals were chosen after LORR had occurred. The time course of the relative power of slow (0.5–1 Hz), delta (1–5 Hz), and alpha (9–14 Hz) frequency bands was extracted. The absolute EEG power of each 3-s bin was normalized to the sum of the power over the entire analysis range (0.5–25 Hz) as previously described (Gui et al., 2021).

The coherence represents the correlation between two signals at a unique frequency (Bokil et al., 2007; Akeju et al., 2014). Coherograms between thalamic LFPs and cortical EEG were computed using the multitaper method (Bokil et al., 2007) based on the continuously recorded electrophysiological signals. Coherence values were calculated for particular epochs (the same as above described for spectral analysis), and the values were averaged for all animals. Jackknife techniques were used to determine 95% confidence interval (CI) of coherence values (Bokil et al., 2007). The parameters for coherence analysis were set as follows: window length, T = 4 s with 0-s overlap; time–oscillation width product, TW = 3; number of tapers, K = 5; and a spectral resolution of 2 W of 1.5 Hz.

### Phase–Amplitude Coupling Analysis

To characterize the coupling between the phase of slow oscillation (0.5–1 Hz) and the amplitude of alpha oscillation (9–14 Hz), we constructed a time-varying phase–amplitude modulogram, which indicated the relative alpha amplitude of a particular phase at each slow oscillation cycle (Mukamel et al., 2011; Purdon et al., 2013). A wavelet packet transform was applied to construct slow and alpha signal narrow oscillations. Then, the Hilbert transform was applied to the signals and low-frequency phase and alpha oscillation amplitudes were computed. Modulogram was computed using the continuously recorded signals. The modulation index (MI) was calculated to quantify the strength of modulation (Mukamel et al., 2011). Signals with the duration of 600 s were selected at 10 min after stabilization of each isoflurane concentration to calculate the total proportions of statistically significant epochs of MI.

### Simulation of Thalamic Networks

A computational model based on the DynaSim MATLAB toolbox (Sherfey et al., 2018) was used to simulate the isoflurane-induced alpha spiking in thalamocortical neurons. The model was based on a thalamic, Hodgkin–Huxley network, which simulates firing frequency of 50 thalamocortical cells (TCs) and 50 reticular single-compartment cells (RE) coupled to each other (the code is available on GitHub, https://github.com/asoplata/propofol-coupling-2017-full) (Soplata et al., 2017). Three paraments were adjusted in this model according to the effects of isoflurane on a single TC neuron: I_GABA-A_ (current of GABA), I_AMPA_ (current of AMPA), and gK_Leak_ (conductance of background potassium channels). The changes in I_GABA-A_ and I_AMPA_ induced by isoflurane (Supplementary Table S1) were demonstrated in previous studies (Jenkins et al., 1999; de Sousa et al., 2000; Sebel et al., 2006). The effect of isoflurane on gK_Leak_ was based on our results of the whole-cell patch-clamping recordings in acute brain slices (Supplementary Figure S1; Supplementary Table S1).

### Statistical Analysis

Processed EEG and LFP data were exported from MATLAB (version 2006a; MathWorks, United States) and analyzed by the GraphPad Prism 8.0 software (GraphPad, United States). Data were presented as means with 95% CI intervals. The sample size of animals was calculated by the test for paired means with the PASS 15 software (NCSS, LLC, Kaysville, UT, United States). By the preliminary test (*n* = 4) on EEG changes of alpha oscillations between baseline and 1.0 MAC_LOM_ isoflurane, it was observed that the isoflurane enhanced relative alpha power from 12.94% [7.95%, 17.92%] to 25.47% [13.17%, 37.77%]. Therefore, the calculated minimal sample size was 8.0 (α = 0.05; power = 0.90), and a sample size of 10 was chosen. Spectral and coherence differences were evaluated by the one-way repeated-measures analysis of variance (ANOVA) with a Greenhouse–Geisser correction. Followed by the repeated-measures ANOVA, Tukey’s post hoc test was used to determine the difference between baseline and anesthetic states, or between various anesthetic depths. For PAC analysis, a permutation test was conducted (Mukamel et al., 2011). Electrophysiologic data were analyzed using the software packages pClamp 10.2 (Molecular Devices, United States), GraphPad Prism 8.0 (GraphPad, United States), and SPSS 22.0 (IBM, United States). The exact statistical methods employed are indicated in the figure legends, and a statistical significance was deemed to be a *p*-value *<* 0.05.

## Results

### Isoflurane-Induced Dynamics of Cortical EEG

**FIGURE 1:**
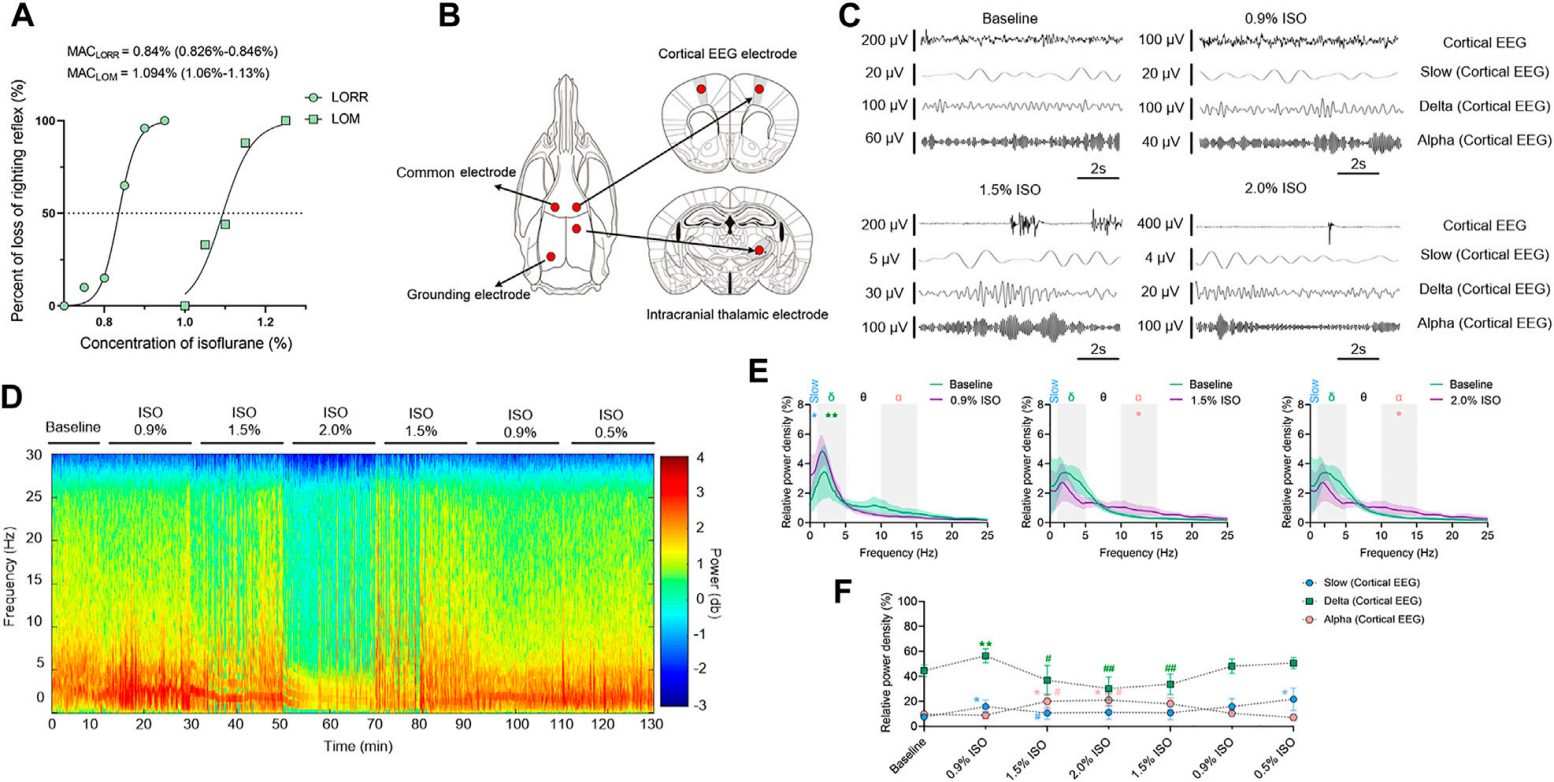
EEG dynamics of the prefrontal cortex under isoflurane anesthesia. **(A)** Concentration–response curves of isoflurane-induced MAC_LORR_ and MAC_LOM_ (*n* = 20/group), MAC_LORR_ = 0.84% [0.83%, 0.85%] and MAC_LOM_ = 1.09% [1.06%, 1.13%]. **(B)** Schematic of recording sites. Red dots represent locations where electrodes implanted. **(C)** Representative raw traces of cortical EEG in slow, delta, and alpha oscillations within the same period. **(D)** A representative spectrogram of cortical EEG. **(E)** A relative power density of 0.9%, 1.5%, and 2.0% isoflurane compared with baseline. Shading indicates 95% CI. **(F)** Total relative power density of cortical EEG in slow, delta, and alpha oscillations. Comparisons are based on the repeated-measures ANOVA for all experiments followed by Tukey’s post hoc test: *p < 0.05 and ** *p* < 0.01 vs. baseline; ^#^ *p* < 0.05 and ^##^ *p* < 0.01 vs. 0.9%. Frequency oscillations: slow (0.5–1 Hz), delta (1–5 Hz), and alpha (9–14 Hz). Values are mean [95% CI]. MAC, minimum alveolar concentration; CI, confidence interval; and ISO, isoflurane.

Concentration–response curves of isoflurane (*n* = 20; Figure 1A) indicated that MAC_LORR_ was 0.84% [0.83%, 0.85%] and MAC_LOM_ was 1.09% [1.06%, 1.13%]. Thus, we defined 0.9% isoflurane as light anesthesia (∼1.0 MAC_LORR_), 1.5% as surgery anesthesia (∼1.3 MAC_LOM_), and 2.0% as deep anesthesia (∼2.0 MAC_LOM_). By the repeated-measures ANOVA, it was found that there were significant differences in the relative power of slow oscillations (_rANOVA_ F _2.035,18.32_ = 3.848, *p* = 0.039; Figure 1F), delta oscillations (_rANOVA_ F _2.09,18.78_ = 11.48, *p* = 0.0005; Figure 1F), and alpha oscillations (_rANOVA_ F _3.15, 28.37_ = 12.16, *p* < 0.0001; Figure 1F) between baseline and anesthetic states. Isoflurane at 0.9% significantly increased the relative power of slow oscillation (*p* = 0.037 by Tukey’s *post hoc* analysis; isoflurane vs. baseline, 15.77% [10.40%, 21.15%] vs. 7.46% [5.92%, 9.01%], *n* = 10; Figure 1E left, Figure 1F and Table 1) and delta oscillation (*p* = 0.0027 by Tukey’s *post hoc* analysis; 0.9% isoflurane *vs.* baseline, 56.43% [50.84%, 62.03%] *vs.* 44.58% [39.96%, 49.20%], *n* = 10; Figure 1E left, Figure 1F and Table 1). 1.5% and 2.0% isoflurane significantly enhanced the relative power of alpha oscillations (*p* = 0.032 by Tukey’s *post hoc* analysis; 1.5% isoflurane *vs.* baseline, 20.02% [14.15%, 25.88%] vs. 9.64% [6.86%, 12.43%], *n* = 10; Figure 1E middle, Figure 1F and Table 1: *p* = 0.029 by Tukey’s *post hoc* analysis; 2.0% isoflurane vs. baseline, 20.92% [15.87%, 25.97%] vs. 9.64% [6.86%, 12.43%], *n* = 10; Figure 1E right, Figure 1F and Table 1). In conclusion, the relative power of cortical slow and delta oscillations increased at 0.9% isoflurane, and the relative power of cortical alpha oscillation was increased at 1.5% and 2.0% isoflurane. As a positive control, when compared to baseline, the cortical alpha power increased during propofol-induced LORR (*p* = 0.007 by a two-tailed paired t-test; propofol vs. baseline, 459.65 [133.71, 785.62] μV^2^/Hz vs. 1548.24 [562.83, 2534.54] μV^2^/Hz, *n* = 10; Figures 4B,C).

**TABLE 1:** Results of cortical EEG and thalamic LFP analysis.

Concentrations of isoflurane		Baseline	0.90%	1.50%	2.00%
Cortical EEG normalized power density (%)	Slow	7.46 [5.92, 9.01]*	15.77 [10.40, 21.15]	10.58 [5.63, 15.53]	11.23 [5.60, 16.86]
	Delta	44.58 [39.96, 49.20]**	56.43 [50.84, 62.03]	36.85 [25.00, 48.69]	30.21 [20.91, 39.52]
	Alpha	9.64 [6.86, 5.75]	8,86 [5.75, 11.98]*	20.02 [14.15, 25.88]*	20.92 [15.87, 25.97]
Thalamic LFP normalized power density (%)	Slow	10.76 [4.44, 17.08]	13.15 [10.13, 16.16]	9.12 [6.19, 12.04]	7.93 [5.41, 10.45]
	Delta	37.39 [32.73, 42.05]	49.18 [42.04, 56.32]*	39.03 [32.85, 45.21]	36.52 [30.13,42.91]
	Alpha	9.20 [7.40, 11.00]	9.71 [6.73, 12.70]	16.38 [12.83, 19.91]**	21.32 [17.26, 25.39]**
Cortical EEG–thalamic LFP coherence index	Slow	0.70 [0.62, 0.77]	0.80 [0.70, 0.89]	0.72 [0.62, 0.83]	0.68 [0.61, 0.74]
	Delta	0.71 [0.63, 0.78]	0.70 [0.62, 0.78]	0.68 [0.58, 0.78]	0.69 [0.62, 0.76]
	Alpha	0.63 [0.58, 0.68]	0.64 [0.56, 0.72]	0.70 [0.63, 0.77]*	0.68 [0.59, 0.77]
Percentage of significant modulation of PAC (%)	Cortex–cortex	11	55	65	35
	Cortex–thalamus	4	50	52	22

*p < 0.05: difference compared with baseline; ** *p* < 0.01: difference compared with baseline; data are given as means [95% CI].

### Isoflurane-Induced Dynamics of Thalamic Local Field Potentials

**FIGURE 2:**
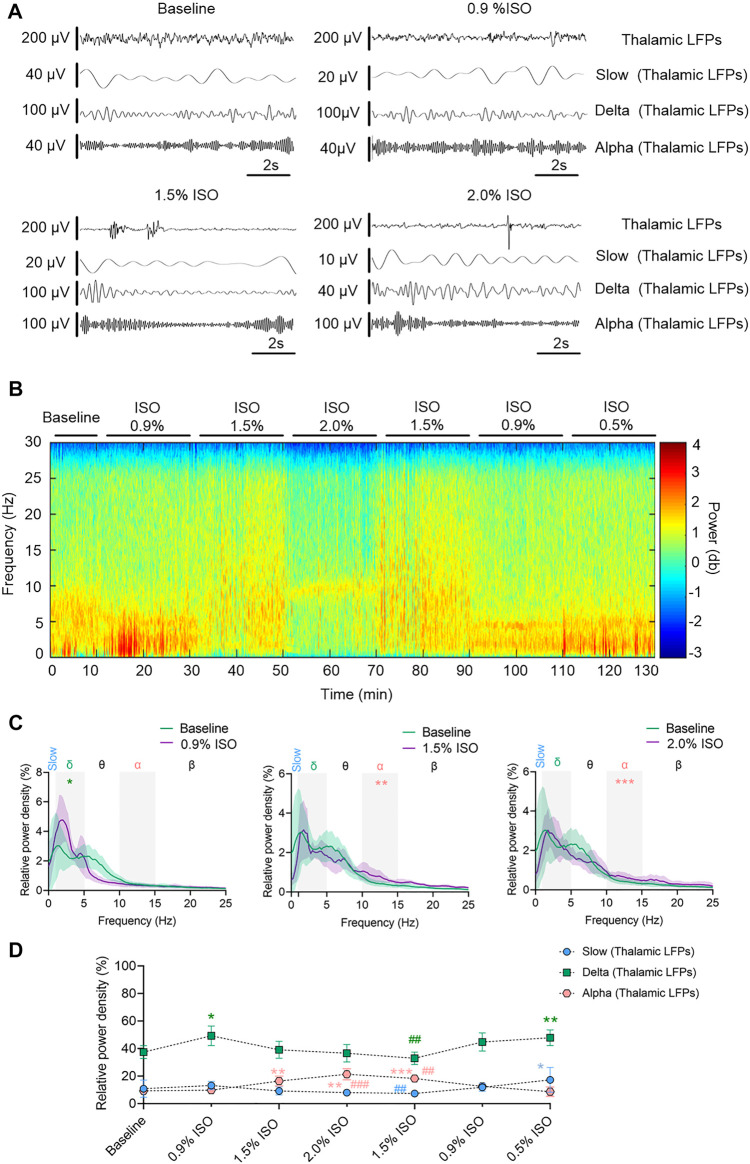
Dynamics of thalamic LFPs under isoflurane anesthesia. **(A)** Representative raw traces of thalamic LFPs in slow, delta, and alpha oscillations within the same period. **(B)** A representative spectrogram of thalamic LFPs. **(C)** A relative power density of 0.9%, 1.5%, and 2.0% isoflurane compared with baseline. Shading indicates 95% CI. **(D)** Total relative power density of thalamic LFPs in slow, delta, and alpha oscillations. Comparisons are based on the repeated-measures ANOVA for all experiments followed by Tukey’s post hoc test: * *p* < 0.05, ** *p* < 0.001, and *** *p* < 0.0001 vs. baseline; ^#^ *p* < 0.05, ^##^ *p* < 0.010, and ^###^ *p* < 0.001 vs. 0.9%. Frequency oscillations: slow (0.5–1 Hz), delta (1–5 Hz), and alpha (9–14 Hz). LFPs, local field potentials; CI, confidence interval; and ISO, isoflurane.

By the repeated-measures ANOVA, it was found that there were no significant differences in the relative power of slow oscillations (_rANOVA_ F _1.64,16.44_ = 3.17, *p* = 0.076; Figure 2D), but there were significant differences in delta (_rANOVA_ F _3.33,33.28_ = 9.10, *p* < 0.0001; Figure 2D) and alpha (_rANOVA_ F _2.65, 26.54_ = 18.17, *p* < 0.0001; Figure 2D) oscillations between baseline and anesthetic states. Isoflurane at 0.9% significantly increased the relative power of delta oscillations (*p* = 0.049 by Tukey’s *post hoc* analysis; 0.9% isoflurane vs. baseline, 49.18% [42.04%, 56.32%] vs. 37.39% [32.73%, 42.05%], *n* = 10; Figure 2C left, Figure 2D and Table 1). Isoflurane at 1.5% and/or 2.0% significantly enhanced the relative power of alpha oscillations (*p* = 0.004 by Tukey’s *post hoc* analysis; 1.5% isoflurane vs. baseline, 16.38% [12.83%, 19.91%] vs. 9.20% [7.40%, 11.00%], *n* = 10; Figure 2C middle, Figure 2D and Table 1; *p* = 0.0007 by Tukey’s *post hoc* analysis; 2.0% isoflurane vs. baseline, 21.32% [17.26%, 25.39%] vs. 9.20% [7.40%, 11.00%], *n* = 10; Figure 2C right, Figure 2D and Table 1). The relative power density of slow, delta, and alpha oscillations over all experimental conditions is shown in Figure 2D. In conclusion, 0.9% isoflurane increased the relative power of thalamic delta oscillation, and 1.5% and 2.0% isoflurane increased the relative power of thalamic alpha oscillation. The dynamics of thalamic LFPs were highly similar to EEG of the prefrontal cortex. As a positive control, the propofol-induced LORR increased the relative thalamic alpha power when compared to baseline (*p* = 0.02 by a two-tailed paired t-test; propofol vs. baseline; 3985.04 [1143.32, 6826.34] μV^2^/Hz vs. 2054.02 [−78.20, 4285.22] μV^2^/Hz, *n* = 10; Figures 4E,F).

### Thalamocortical Synchronization Maintained With Increased Depth of Isoflurane Anesthesia

**FIGURE 3:**
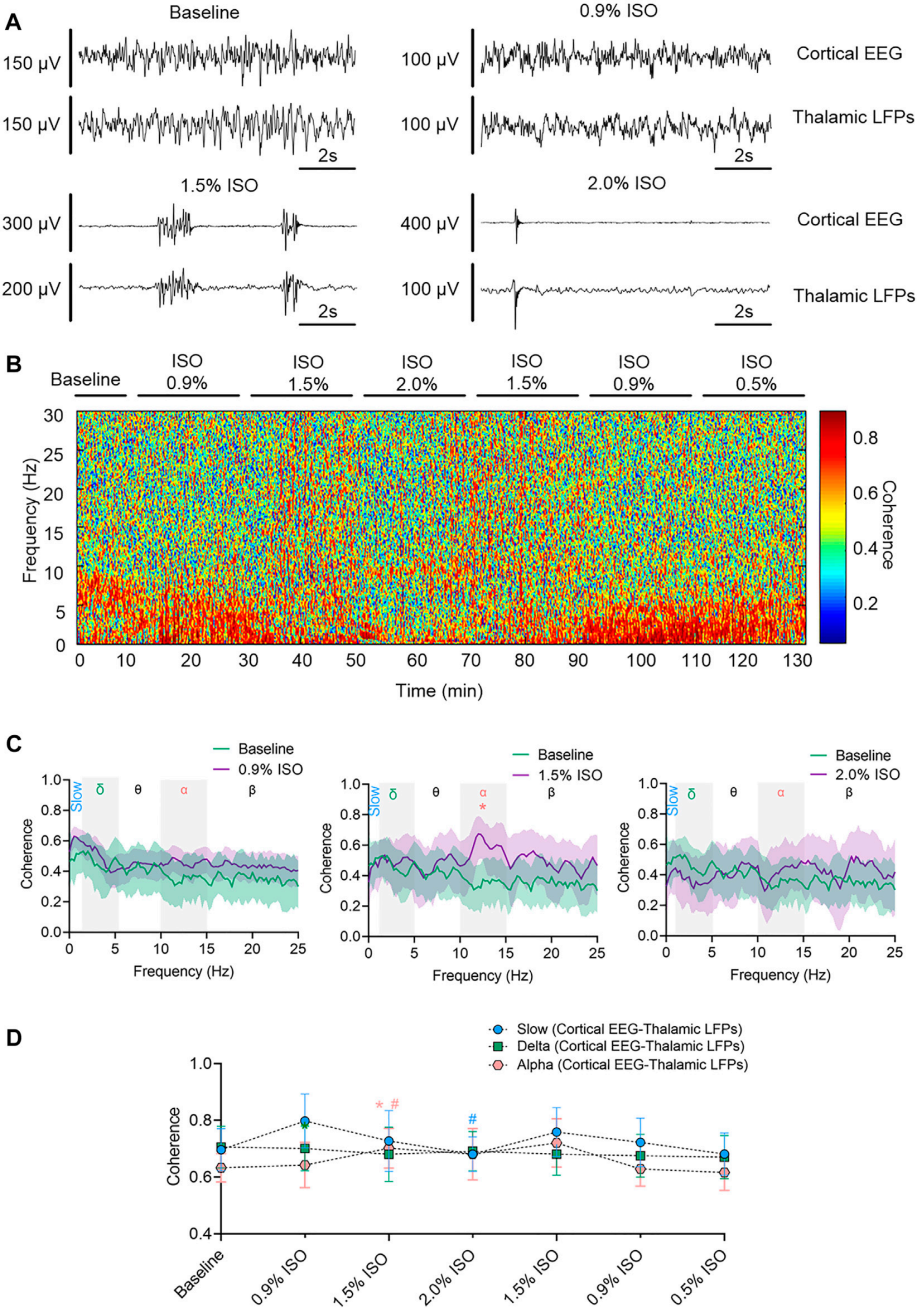
Thalamocortical synchronization under isoflurane anesthesia. **(A)** Representative raw traces of cortical EEG (upper) and thalamic LFPs (lower) within the same period. **(B)** Representative coherogram between cortical EEG and thalamic LFPs. **(C)** Coherence between cortical EEG and thalamic LFPs. Shading indicates 95% CI. **(D)** Coherence between thalamic LFPs and cortex EEG. Comparisons are based on the repeated-measures ANOVA for all experiments followed by Tukey’s post hoc test: * *p* < 0.05 vs. baseline. Frequency oscillations: slow (0.5–1 Hz), delta (1–5 Hz), and alpha (9–14 Hz). LFPs, local field potentials; CI, confidence interval; and ISO, isoflurane.

**FIGURE 4:**
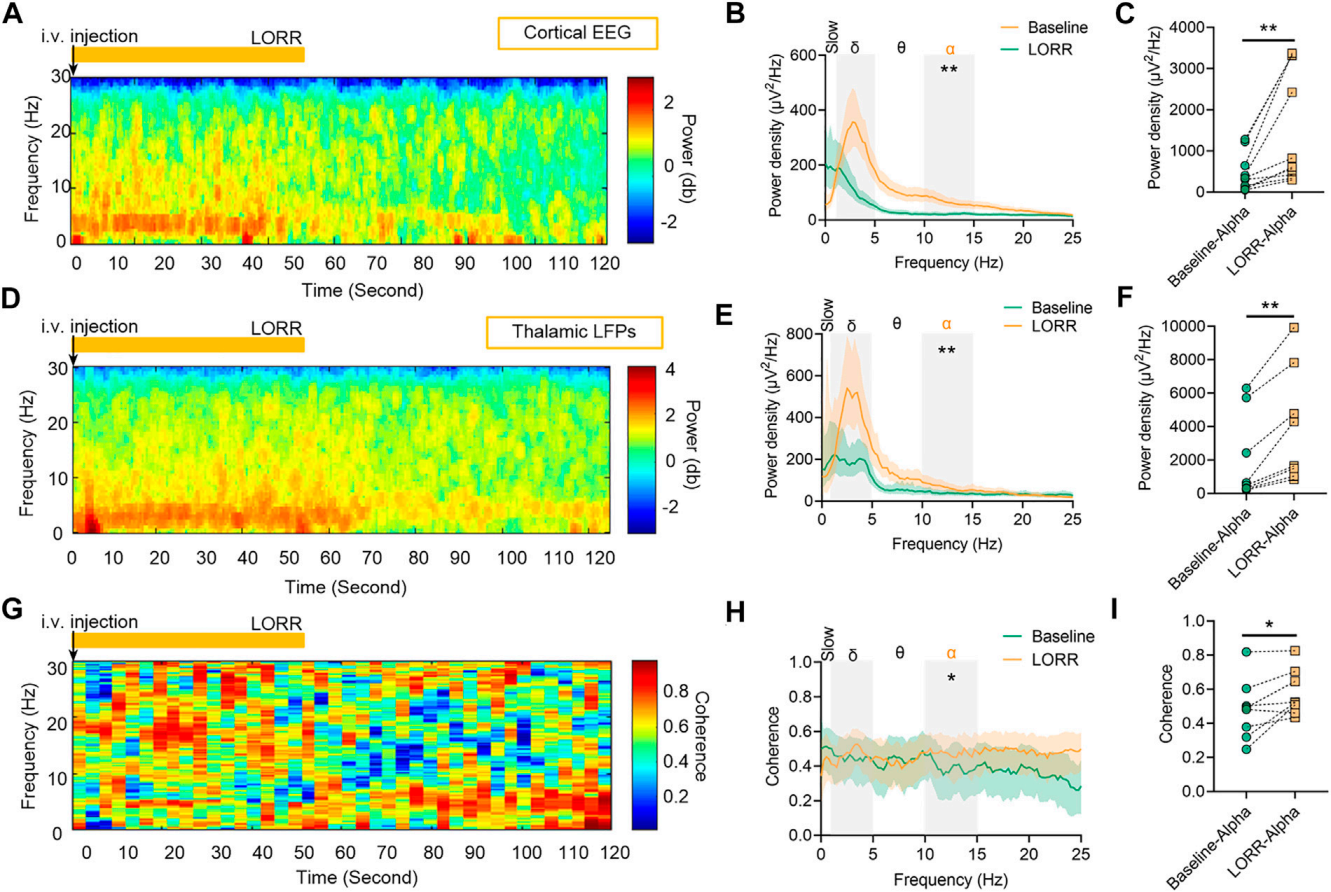
Dynamics of cortical EEG and thalamic local field potentials (LFPs) under propofol anesthesia. **(A)** Representative spectrograms of cortical EEG and **(D)** thalamic LFPs. Propofol dosing starts at *t* = 0, and the vertical orange lines mark behavioral events (LORR). **(B)** Relative power density of cortical EEG and **(E)** thalamic LFPs. **(C)** Shading indicates 95% CI. The power density of cortical EEG and **(F)** thalamic LFPs in alpha oscillations. **(G)** Spectrogram of coherence between thalamic LFPs and cortical EEG. **(H)** Coherence between thalamic LFPs and cortical EEG. Shading indicates 95% CI. **(I)** Coherence of alpha oscillations between thalamic LFPs and cortical EEG. Comparisons are based on a two-tailed paired t-test: *p < 0.05 and **p < 0.01. Frequency oscillations: slow (0.5–1 Hz), delta (1–5 Hz), and alpha (9–14 Hz). LFPs, local field potentials; and CI, confidence interval.

By the repeated-measures ANOVA, it was found that there was no statistical difference in coherence between cortical EEG and thalamic LFPs in slow band (_rANOVA_ F _3.34,33.38_ = 1.83, *p* = 0.16; Figures 3C,D) or delta band (_rANOVA_ F _3.45,34.53_ = 0.38, *p* = 0.80; Figures 3C,D), but there was a significant difference in alpha band (_rANOVA_ F _2.70, 26.95_ = 4.15, *p* = 0.018; Figures 3C,D). Isoflurane at 1.5% enhanced the coherence of alpha oscillations between cortical EEG and thalamic LFPs (*p* = 0.028 by Tukey’s *post hoc* analysis; 1.5% isoflurane vs. baseline, 0.70 [0.63, 0.77] vs. 0.63 [0.58, 0.68], *n* = 10; Figure 3C middle, Figure 3D and Table 1). As a positive control, the propofol-induced LORR increased the coherence of alpha oscillation between cortical EEG and thalamic LFPs (*p* = 0.02 by a two-tailed paired t-test; propofol vs. baseline, 0.58 [0.47, 0.69] vs. 0.48 [0.33, 0.62], *n* = 10; Figure 4H,I).

### The Slow–Alpha PAC Is Significantly Correlated Under 1.5% Isoflurane but Disrupted Under 2.0% Isoflurane

**FIGURE 5:**
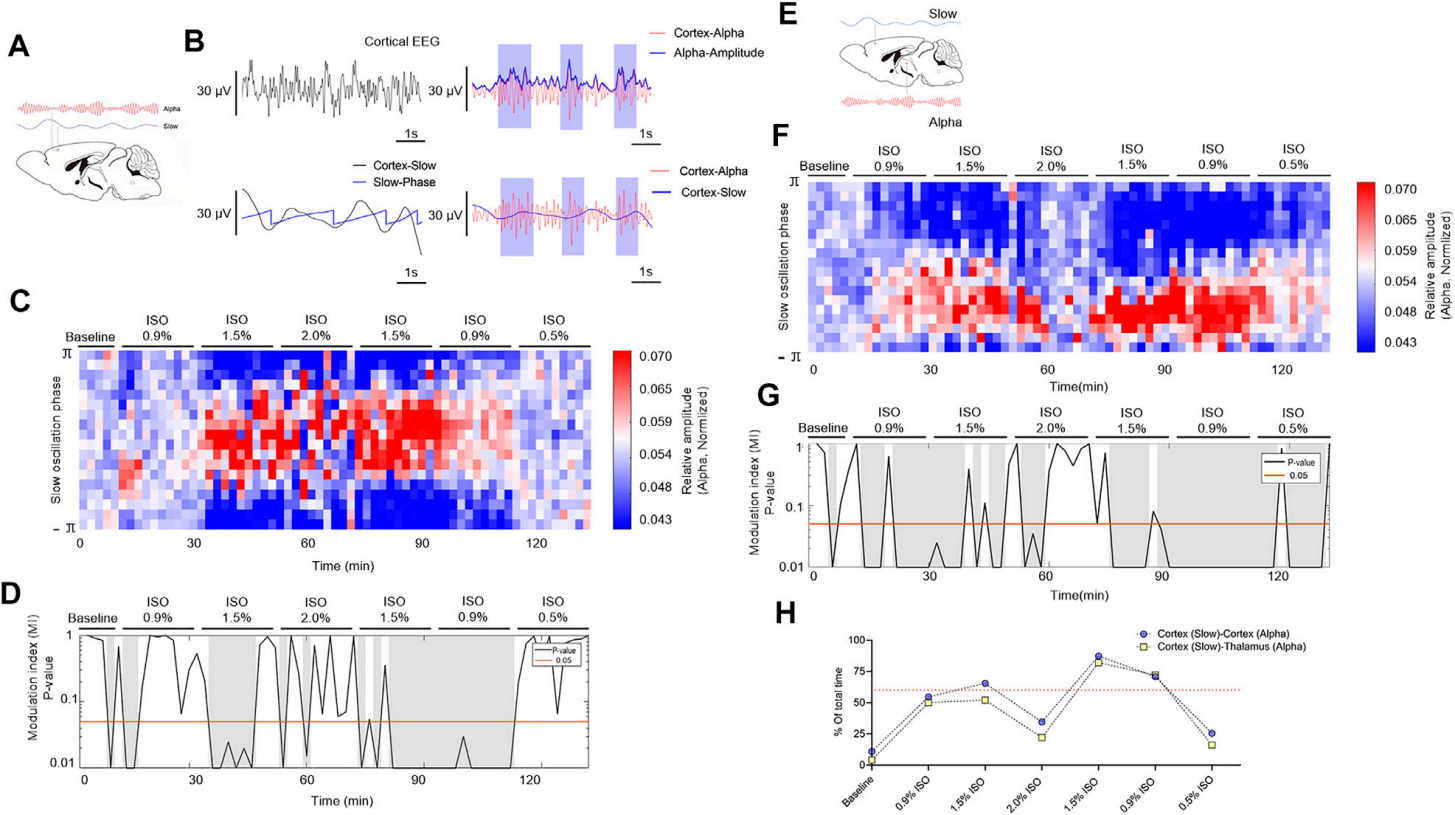
PAC in cortex and thalamocortical networks. **(A)** Schematic cortex slow (blue line) and cortex alpha (red line) oscillations. **(B)** A sample of PAC analysis process. **(C)** The phase–amplitude histogram showing the relationship between the cortical slow phase (y-axis) and cortical alpha amplitude (color map). **(D)** *p*-Value of the modulation index (MI), determined by a shuffle control procedure; gray shading indicates statistically significant (*p* < 0.05) epochs. **(E)** Schematic of PAC between cortex slow (blue line) and thalamic alpha (red line). **(F)** The phase–amplitude histogram showing the relationship between the cortical slow phase (y-axis) and thalamic alpha amplitude (color map). **(G)** *p*-Value of the modulation index, determined by a shuffle control procedure; gray shading indicates statistically significant (*p* < 0.05) epochs. **(H)**The total proportions of gray shadings **(D,G)** of all subjects. The dotted line shows the proportions at 60%. PAC, phase–amplitude coupling; and ISO, isoflurane.

The phase and amplitude of slow and alpha oscillations filtered from cortical EEG (Figure 5A) were extracted by the Hilbert transform (Figure 5B) and were calculated by the time-dependent modulogram (Figure 5C). A significant slow–alpha PAC within the cortex was observed under 1.5% isoflurane (*p <* 0.05 in 65.5 percent of total duration of 220 min by a permutation test; Figures 5D–H and Table 1), while 2.0% isoflurane disrupted the PAC (*p* < 0.05 in 34.5 percent of total duration of 220 min by a permutation test; Figures 5D–H and Table 1). The cortical alpha amplitude and thalamic slow phase (Figure 5F) were also calculated using the time-dependent modulogram. A significant slow–alpha phase–amplitude modulation between the cortex and the thalamus was observed under 1.5% isoflurane (*p* < 0.05 in 58.0 percent of total duration of 220 min by a permutation test; Figures 5G,H), while 2.0% isoflurane disrupted the PAC (*p* < 0.05 in 22.1 percent of total duration of 220 min by a permutation test; Figures 5G,H).

### Effects of Isoflurane on Potassium Leak Conductance in Thalamic TC Neurons

The effects of isoflurane on K_Leak_ current were measured by the whole-cell patch-clamping recordings in TC (Supplementary Figure S2A). TC neurons displayed a weakly rectifying current–voltage profile, and reversal potential was near E_K_ (approximately –90 mV), as expected for a K_Leak_ current (Supplementary Figure S2B) (Lazarenko et al., 2010). An isoflurane-enhanced K_Leak_ current (Supplementary Figure S2C, top) was associated with a net increase in conductance (Supplementary Figure S2C, bottom). 0.26–0.30 mM isoflurane solution (∼1.0 MAC_LOM_) increased K_Leak_ conductance (gK_Leak_) (_rANOVA_ F_1.5, 7.7_ = 8.4, *p* = 0.01; *p* = 0.009 by Tukey’s *post hoc* analysis; control *vs.* 1.0 MAC_LOM_ isoflurane, 30.40 nS [4.48 nS, 56.32 nS] vs. 39.04 nS [4.52 nS, 73.56 nS], *n* = 7; Supplementary Figure S2D). 0.42–0.50 mM isoflurane solution (∼1.5 MAC_LOM_) increased K_Leak_ conductance (_rANOVA_ F_1.2, 8.7_ = 8.4, *p* = 0.002; *p* = 0.008 by Tukey’s *post hoc* analysis; control vs. 1.5 MAC_LOM_ isoflurane, 27.03 nS [14.10 nS, 39.94 nS] vs. 39.29 nS [26.54 nS, 52.04 nS], *n* = 7; Supplementary Figure S2E). Therefore, 0.26–0.30 mM (∼1.0 MAC_LOM_) and 0.42–0.50 mM (∼1.5 MAC_LOM_) isoflurane solution enhanced gK_Leak_ by 25.86% [10.91%, 40.81%] and 47.10% [27.98%, 66.22%], respectively (Supplementary Figure S2F).

### Effects of Isoflurane on Multiple Molecular Targets in the Thalamic Network Contribute to Isoflurane-Induced Alpha Spiking in TC Neurons in a Computational Model

**FIGURE 6:**
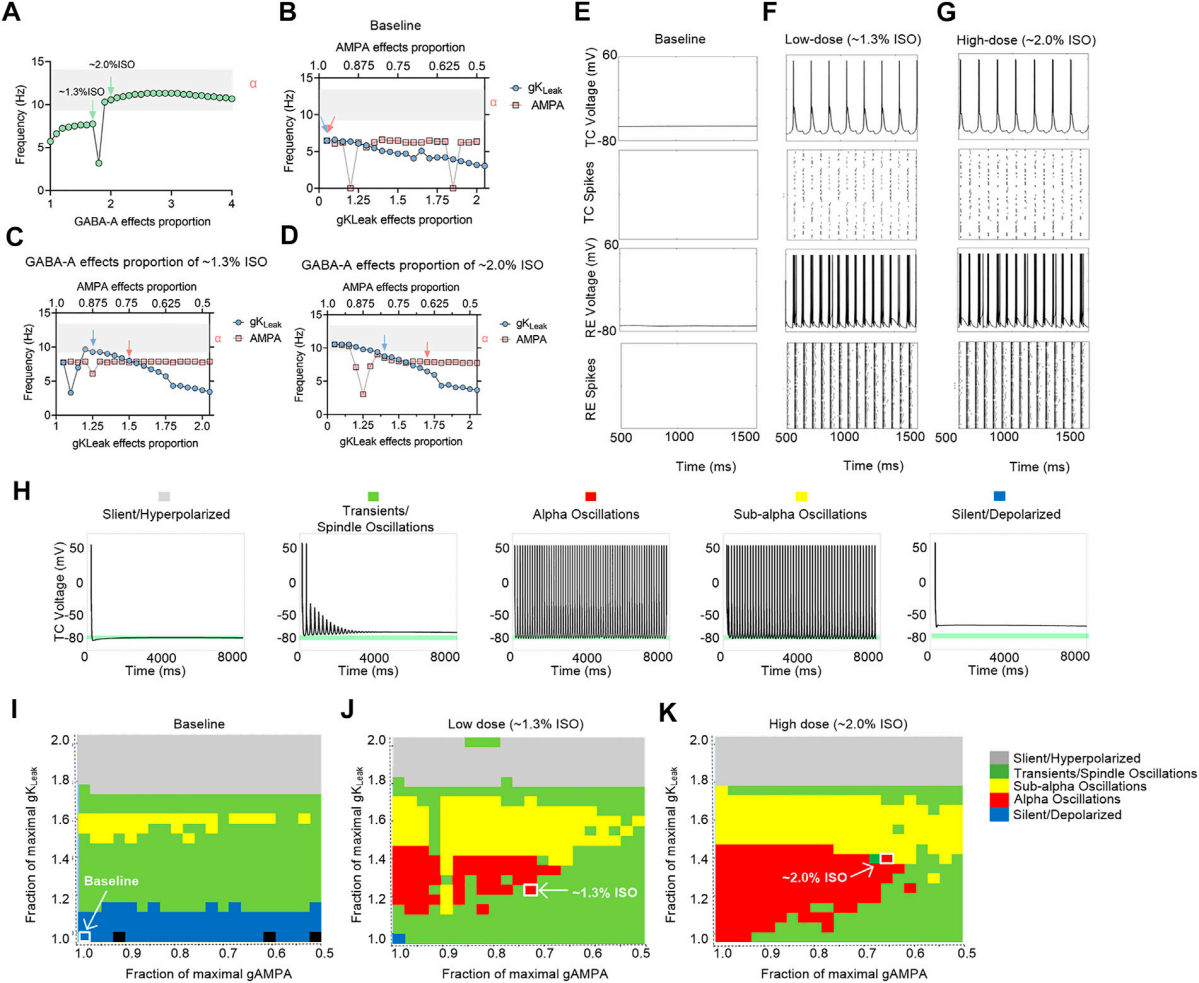
Computational simulation of thalamic networks indicates that the overall effects of isoflurane on AMPA, GABA_A_, and K_Leak_ may lead to thalamic alpha oscillations. **(A)** Frequency of the TC spike increases to alpha oscillations (gray shadow) along with the potentiation of GABA_A_. The maximal AMPA current decreases (red dotted trace) and gK_Leak_ increases (blue dotted trace): **(B)** no GABA_A_ current potentiation; **(C)** GABA_A_ current potentiation of ∼1.3% isoflurane; **(D)** GABA_A_ current potentiation of ∼2.0% isoflurane; **(E)** under baseline (no isoflurane potentiation of any molecular targets) conditions; **(F)** ∼1.3% isoflurane potentiation of GABA_A_ current, AMPA current, and gK_Leak_; and **(G)** ∼2.0% isoflurane potentiation of those; representative voltage traces and spike rastergrams of TC and RE neurons; **(H)** representative TC cell voltage trace during silent, hyperpolarized stimulation, transients, spindling oscillation simulation, subalpha oscillation simulation, and silent, depolarized simulation. Behavioral regimes of thalamic simulations across different GABA_A_ potentiation levels of the gK_Leak_—AMPA current plane: **(I)** baseline; **(J)** 1.3% isoflurane; and **(K)** 2.0% isoflurane. Each simulation is represented by a pixel and colored according to its manually classified behavioral regime. Arrows indicate the alpha oscillations corresponding to the parameters in Supplementary Table S1. ISO, isoflurane.

To investigate the preliminary mechanism of how isoflurane induced alpha spiking in the thalamic network, a *in silico* thalamic model was used to simulate the effects of isoflurane on thalamic rhythms (Soplata et al., 2017). Briefly, AMPA, GABA_A_, and K_Leak_ were the main molecular targets in this model. Therefore, the effects of isoflurane on AMPA, GABA_A_, and K_Leak_ were applied in simulation. The effects of isoflurane on AMPA (de Sousa et al., 2000) and GABA_A_ (Sebel et al., 2006) were demonstrated in previous studies. The effect of isoflurane on gK_Leak_ was based on our results of the whole-cell patch-clamping recordings on acute brain slices (Supplementary Figure S2). When the potentiation of GABA_A_ was up to ∼twofold more than the baseline level, the frequency of the TC spikes increased to alpha oscillations (Figure 6A). If the contribution of GABA_A_ was ignored, the TC spikes decreased along with the potentiation of gK_Leak_, while the inhibition of I_AMPA_ slightly modulated the network frequency (Figure 6B). When the potentiation of I_GABA-A_ was adjusted to the levels of 1.3% isoflurane (∼1.7-fold potentiation) (Figure 6C) and 2.0% isoflurane (∼twofold potentiation) (Figure 6D), respectively, the TC spikes decreased with the potentiation of gK_Leak_ (Figure 6B) and were nearly stable with the inhibition of I_AMPA_.

On the cellular level, thalamic TC neurons did not display any intrinsic oscillatory activity at the baseline level (Figure 6E). If the overall modulations of isoflurane on GABA_A_, gK_Leak_, and AMPA were applied simultaneously, isoflurane at both ∼1.3% (Figure 6F) and ∼2.0% (Figure 6G) induced persistent alpha firing of TC neurons. On the network level, no alpha oscillations were induced in a thalamic rhythm at the baseline condition (Figure 6I). With the potentiation of GABA_A_ effect under ∼1.3% (Figure 6J) and ∼2.0% isoflurane (Figure 6K), respectively, alpha oscillations were induced along with the modulation of gK_Leak_ and I_AMPA_. Isoflurane at both ∼1.3% (Figure 6J) and ∼2.0% (Figure 6K) induced persistent alpha oscillations in thalamic rhythm. In summary, the overall modulation of isoflurane on I_AMPA_, I_GABA-A_ current, and gK_Leak_ may contribute to alpha spiking of TC neurons under surgery and/or deep anesthesia under isoflurane, which may involve in a thalamic alpha rhythm.

## Discussion

In the present study, we observed cortical EEG and thalamic LFPs under isoflurane in mice, which shows differential patterns of slow, delta, and alpha oscillations when compared with propofol. By combining the electrophysiological results and a computational model (Soplata et al., 2017), we simulated isoflurane-induced alpha spiking in TC neurons, which reveals the molecular targets and thalamic network mechanisms of isoflurane-induced alpha spiking in the thalamic network. Considering there are still no such standards for tracking brain states under volatile anesthesia (Purdon et al., 2013; Malekmohammadi et al., 2019), our results may provide neurophysiological signatures for monitoring isoflurane anesthesia at various depths.

There have still been some debates on the association between consciousness and EEG signatures. Some studies demonstrated that the level of consciousness may be dissociated from cortical connectivity, oscillations, and dynamics. These previous studies analyzed the changes in functional cortical gamma connectivity (25–155 Hz), slow oscillations (0.5–1 Hz), and complexity (Pal et al., 2018; Pal et al., 2020), while the association between consciousness and coherence or connectivity of other frequency bands (such as alpha) has been proved by numerous previous studies (Cimenser et al., 2011; Purdon et al., 2015; Flores et al., 2017; Guidera et al., 2017). Such debates may be also raised from the differential definitions of consciousness levels with different experimental designs. Although the debate exists, the analysis of the EEG characteristics has been established as a basic method for monitoring anesthetic depth currently used in the clinic, such as Bispectral Index (Bonhomme et al., 2000; Meuret et al., 2000) and Patient State Index (Jones et al., 2021; Obara et al., 2021). Instead of revealing the EEG changes with different consciousness states, our present study was designed to explore the electrophysiological changes with increased concentration of isoflurane.

Isoflurane induces slow, delta, and alpha oscillations with different patterns, when compared to propofol. Based on the previous study (Flores et al., 2017) and our results, propofol-induced cortical and thalamic alpha oscillations start to increase before LORR, and cortical alpha oscillations further increase at LOM. In slow and delta oscillations, both cortical EEG and thalamic LFPs induced by propofol show a significant enhancement when LOM had occurred (Brown et al., 2010). However, in the present study, under 0.9% isoflurane (∼MAC_LORR_), both cortical EEG and thalamic LFPs showed an enhancement in slow and delta bands but no change in the alpha band. As isoflurane concentration elevated to 1.5% (∼MAC_LOM_) or even higher (2.0%), both cortical and thalamic alpha oscillations exhibited a significant increase which paired with decreased slow–delta oscillation. These results are similar to those of some previous studies, in which sevoflurane was administered and which reported no change in the alpha band when LORR (in rodents) or LOC (loss of consciousness, in humans) had occurred (McCallum et al., 2013; Akeju et al., 2014; Purdon et al., 2015; Guidera et al., 2017).

Here, the thalamocortical coherence of alpha oscillation only exhibits a significant increase under 1.5% isoflurane (surgical anesthesia, ∼MAC_LOM_), while the coherence of slow and delta oscillations is maintained at a baseline level. The coherence of alpha oscillation induced by propofol increases significantly when LORR had occurred. The hypothesis that increasing order in neural networks may contribute to unconsciousness (Tononi, 2008; Chauvette et al., 2011; Alonso et al., 2014) is partly supported by our findings. However, of note, the coherence observed in distant brain regions (separated by millimeters) varied in different studies. For example, at the doses of sevoflurane sufficient to maintain LORR, slow–delta oscillations in distant brain regions are highly coherent (Guidera et al., 2017). Also, asynchronous slow oscillations have been observed in distant regions of the temporal cortex in propofol-anesthetized humans (Silva et al., 2010; Lewis et al., 2012). We believe that both synchronous and asynchronous oscillations may play functional roles in anesthetic-induced unconsciousness, but within different brain regions and networks, respectively.

Anesthesia-induced PAC has been explored previously (Molaee-Ardekani et al., 2007; He and Raichle, 2009; Breshears et al., 2010; Murphy et al., 2011). Propofol-induced PAC (cortical slow phase and thalamic alpha amplitude) in humans shows the existence of two distinct patterns: the trough-max pattern and the peak-max pattern (Purdon et al., 2013). The trough-max pattern offers a predictor of recovery of consciousness (ROC), whereas the peak-max pattern provides a signature of profound unconsciousness (Purdon et al., 2013). Since peak-max PAC disables responsiveness even under noxious stimuli (Gaskell et al., 2017), it is believed that the phase–amplitude modulation can be used to improve anesthesia depth monitoring (Ching et al., 2010; Soplata et al., 2017). However, the isoflurane-induced PAC modulation only reveals a unique pattern, which maintains to be unchanged throughout different concentrations. According to a computational model, two PAC modulation patterns induced by propofol are induced by GABA_A_ potentiation (Soplata et al., 2017). Although volatile anesthetics also induce actions on GABA_A_ receptor, the isoflurane-induced PAC modulation is not related to various behavioral endpoints (LOC, ROC, or LOM). Therefore, for volatile anesthetics, the value of PAC modulations in monitoring anesthetic depth still needs further studies.

These different EEG patterns induced by isoflurane may be underlined by different molecular mechanisms. Compared with propofol, a relatively pure GABA_A_ receptor modulator, an enhanced GABAergic inhibition is likely a primary but not the only mechanism of inhalation anesthetics. Isoflurane has multiple targets including GABA_A_, AMPA, and K_Leak_ primarily in the thalamus (Hemmings et al., 2005; Franks, 2008). Electrophysiology study also indicated that the isoflurane enhanced the inhibition of thalamic neurons through GABA_A_-dependent and GABA_A_-independent mechanisms (Ying et al., 2009). To investigate whether the isoflurane-induced alpha oscillation shares the same thalamocortical networks, we take the advantage of a computational model (Soplata et al., 2017). The whole-cell patch-clamping results provide us to what extent did isoflurane change I_GABA-A_, I_AMPA_, and gK_Leak_. In this study, the effect of isoflurane on K_Leak_ was investigated on acute brain slices under the conditions such as blocked voltage-gated sodium channels (Na_v_), and fast excitatory (glutamate) and inhibitory transmitters (GABA, glycine) by adding 1 μM tetrodotoxin, 10 μM CNQX, 10 μM bicuculline, and 30 μM strychnine in a perfusion solution (as described in the Supplementary Methods). This is important because this model stimulated the interaction between multiple molecular targets (AMPA, GABA_A_, and K_Leak_); therefore, the effect of isoflurane on individual targets that are used for the model is only valid when the modulatory effects are fully independent. Otherwise, the effects of isoflurane on individual target will be repetitively calculated. By changing paraments in the model based on these whole-cell patch-clamping results, we successfully simulated alpha oscillation of TC cells induced by isoflurane, which consisted of thalamic LFPs. This finding supported our hypothesis that both propofol and inhalation anesthetics could induce an alpha oscillation within the same circuits linking the thalamus and the frontal cortex.

Based on the experimental design of *in vivo* recordings here, it is a bipolar montage system that records the voltage difference between the recording and common electrodes. The distance between the cortical EEG electrode and the common electrode is very far (∼3 mm) within the frontal cortex, and thalamic electrode and common electrode are not in the same brain region. Therefore, the recorded signals in the present study does not display the absolute activity within the small site at the electrode location. For this reason, literatures from previous human and nonhuman studies, which used a single-site recording (Flores et al., 2017; Guidera et al., 2017; Malekmohammadi et al., 2019), may be not directly comparable to the findings from this study. Some signatures reported here should be careful when compared to other studies. Of note, the primary purpose of the present study, that is, to investigate the synchronization between the cortex and the thalamus with various depths of isoflurane, is still significant because the same common electrode was used for both cortical EEG electrode and thalamic electrode. Therefore, the relationship between the cortex and the thalamus (coherence and PAC) can be primarily analyzed by subtracting the same reference signal.

There were still some minor limitations in this study. First, we performed this study with a two-channel EEG recording system. We could only record one cortical and one thalamic area at the same time. Future studies can record from multiple cortical and thalamic areas to characterize whether they participate in these dynamics. Second, because of the different anatomical structure, there were still many apparent dissimilarities in EEG dynamics in rodents and humans under general anesthesia; therefore, the EEG signatures under isoflurane may not be directly applied in humans. Third, the model used here contains 50 TC and 50 RE Hodgkin–Huxley single-compartment cells connected all to all, which is too small to fully simulate thalamic LFPs. However, this model is used to stimulate the firing frequency of thalamic TC cells with or without general anesthetics, which can primarily investigate the molecular targets of volatile anesthetics in the thalamic network. In addition, 50 thalamic cells may be enough to connect to roughly 8000 cortical cells (O'Kusky and Colonnier, 1982); therefore, alpha frequency firing of thalamic TC cells under volatile anesthetics may at least partly contribute to thalamic alpha LFPs (sum of extracellular spiking) and ascending projection of the corticothalamic network.

In summary, our present study demonstrates that isoflurane can induce slow, delta, and alpha oscillations with differential patterns compared with propofol. These specific signatures under isoflurane-induced anesthesia can help to build standards for tracking brain states under general anesthesia, which can prevent intraoperative awareness, postoperative delirium, or postoperative cognitive dysfunction. By combining electrophysiological experiments and modeling paradigms, the results may provide a new approach in relating drug-specific EEG signatures to molecular mechanisms. The increased understanding of anesthetic neurophysiology will provide new insights into brain function and altered states of consciousness or arousal under general anesthesia.

## Data Availability

The original contributions presented in the study are included in the article/Supplementary Material, further inquiries can be directed to the corresponding authors.
