# Supplementary material for: Signatures of Thalamocortical Alpha Oscillations and Synchronization With Increased Anesthetic Depths Under Isoflurane
Source: Front Pharmacol. 2022 Jun 3;13:887981. doi: 10.3389/fphar.2022.887981 (PMC9204038; doi:10.3389/fphar.2022.887981)
Supplement: Supplementary file 1 [file DataSheet1.docx]

**SUPPLEMENT METHODS AND MATERIAL**

**Preparation of acute brain slices**

Brain slices were prepared as previously described (Yang et al., 2020). Briefly, 4-week-old C57 mice were decapitated under ketamine/xylazine anesthesia. Transverse brain slices (200 μm) containing VPL (ventroposterolateral thalamic nuclei) were cut on a vibratome (VT1000 A; Leica, USA) in ice-cold dissecting solution containing (in mM) 260 sucrose, 3 KCl, 5 MgCl_2_, 1 CaCl_2_, 1.25 NaH_2_PO_4_, 26 NaHCO_3_, 10 glucose, and 1 kynurenic acid. Then the slices were incubated for 40-50 min at 37°C in incubation solution contained the following (in mM): 130 NaCl, 3 KCl, 2 MgCl_2_, 2 CaCl_2_, 1.25 NaH_2_PO_4_, 26 NaHCO_3_ and 10 glucoses. Both the dissecting and incubation solutions were equilibrated with 95% O_2_ and 5% CO_2_ (pH 7.35). Two to three neurons were recorded on each brain slices, and 5 mice were included in the study.

**Whole****-cell patch-clamping recordings**

Whole-cell recordings were performed on thalamocortical cells (TC) neurons of VPL at room temperature (21°C-23°C) as previously described (Landisman and Connors, 2007; Haidarliu et al., 2008). For brief, each brain slice was deposited in a recording chamber and submerged by external bath solution (~2 ml/min). And 1 μM tetrodotoxin, 10 μM CNQX, 10 μM bicuculline and 30 μM strychnine was added in the bath solution to block voltage-gated sodium channels (Na_v_), inhibit fast excitatory (glutamate) and inhibitory transmitters (GABA, glycine) respectively. Electrophysiological measurements were taken with an Axopatch 700B amplifier and Digidata1440 digitizer, then the data was fed to a computer running pClamp 10.2 (Molecular Devices, USA). Holding currents and conductance were monitored over time by delivering −60 mV voltage steps every 170 ms. To characterize anesthetic-sensitive currents, we obtained steady-state current-voltage (I-V) relationships from a holding potential of –60 mV by applying voltage steps (from –30 to –120 mV, in 10 mV increments) under control conditions and in the presence of isoflurane (0.26-0.28 mM, ~1.0 MAC_LOM_ and 0.39-0.42 mM, ~1.5 MAC_LOM_). Isoflurane-sensitive I-V curves were obtained in each cell by subtraction, and the data from individual cells was averaged.

**Table S1.** Parameters for the simulation of thalamic network in silico

| Parameters | Low-dose (~1.3% ISO) | | High-dose (~2.0% ISO) | |
| --- | --- | --- | --- | --- |
|  | Electrophysiology  (% change) | Simulation  (% change) | Electrophysiology  (% change) | Simulation  (% change) |
| I_GABA-A_ | 68.7 ± 10.7 (Jenkins et al., 1999; de Sousa et al., 2000; Sebel et al., 2006) | 70.0 | ~100.0 ± 10.0 (Jenkins et al., 1999; de Sousa et al., 2000; Sebel et al., 2006) | 100.0 |
| I_AMPA_ | –27.8 ± 6.7 (de Sousa et al., 2000) | –27.5 | ~–38.0 ± 5.0 (de Sousa et al., 2000) | –35.0 |
| gK_Leak_ | 25.86 [10.91, 40.81] | 25 | 47.10 [27.98, 66.22] | 40.0 |

Data of I_GABA-A_ and I_AMPA_ were presented as means ± SD. Data of gK_Leak_ were presented as means with 95% CI values. I_GABA-A_, current of GABA_A_; I_AMPA_, current of AMPA; gK_Leak_, conductance of background potassium channels; ISO, isoflurane.


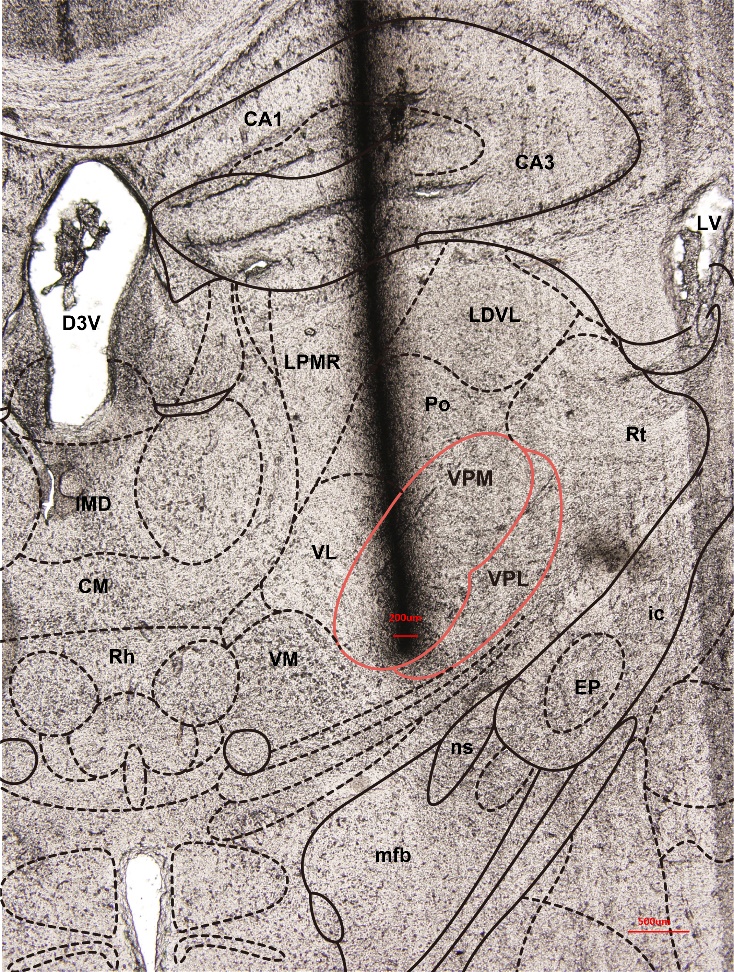


**Fig. S1:** Histological localization of electrodes targeting VPM/VPL of thalamic nuclei. Example of electrode locations and and its size (200 μm) in a coronal slice.


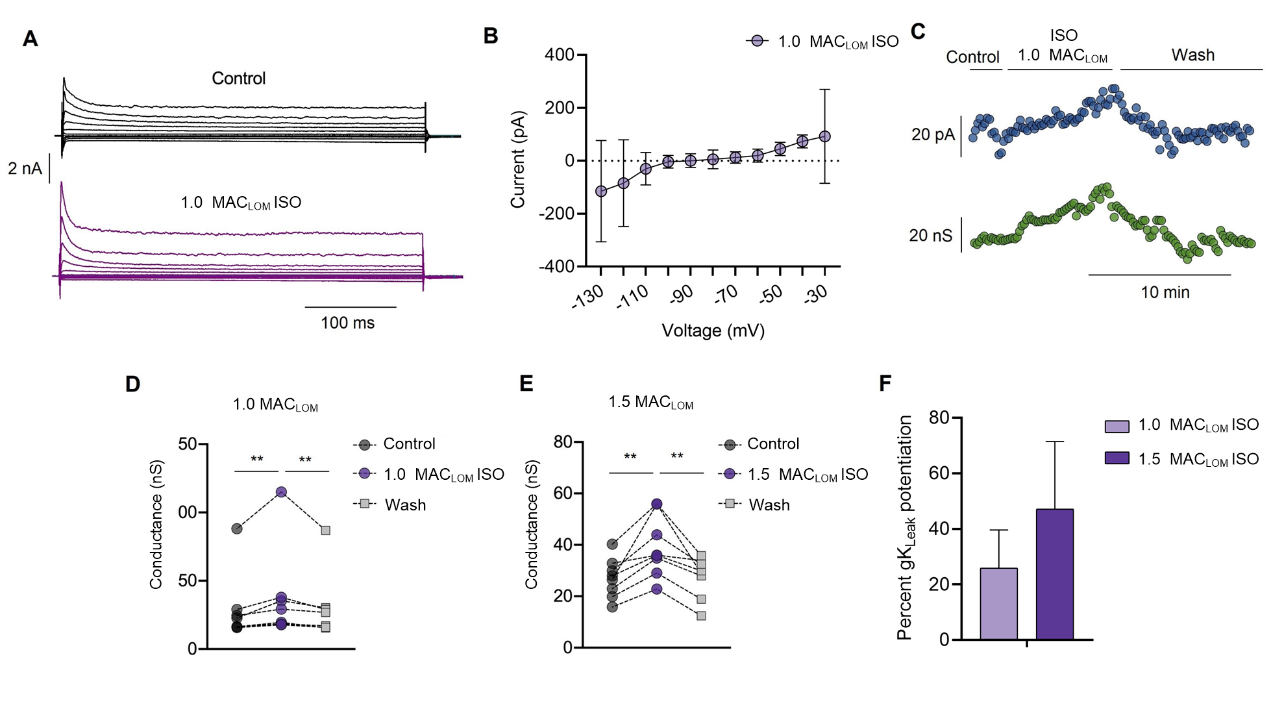


**Fig. S2:** Effects of isoflurane on leak potassium conductance (K_Leak_) in thalamic TC neurons. (A) Representative I-V curves recorded from –130 to –30 mV under control or ~1.0 MAC_LOM_ isoflurane; (B) Averaged I-V relationship of the isoflurane- potentiated current (isoflurane – control) in cells, for which the isoflurane-induced inward current was associated with enhanced (n = 8). The overlaid curve is a fit of the Goldman-Hodgkin-Katz equation for current of K_Leak_; (C) Whole-cell voltage-clamp recordings indicate that ~1.0 MAC_LOM_ isoflurane enhances the holding currents (top) and holding conductance (bottom); (D-E) The summary data indicates that ~1.0 MAC_LOM_ (D) and ~1.5 MAC_LOM_ (E) isoflurane increases holding conductance. Data were presented as means with 95% CI values. Comparisons are based on repeated-measured ANOVA analysis for all experimental followed by turkey’s *post hoc* test: * *P* < 0.05 *vs* baseline: *P < 0.05; **P < 0.01; (F) Concentration-dependent effects of isoflurane on the conductance of K_Leak_ (n = 8). TC, thalamocortical cell; CI, confidence interval; ISO, isoflurane.

**References:**

de Sousa, Sara L.M., Dickinson, R., Lieb, William R., and Franks, Nicholas P. (2000). Contrasting Synaptic Actions of the Inhalational General Anesthetics Isoflurane and Xenon. *Anesthesiology* 92(4)**,** 1055-1066. doi: 10.1097/00000542-200004000-00024.

Haidarliu, S., Yu, C., Rubin, N., and Ahissar, E. (2008). Lemniscal and Extralemniscal Compartments in the VPM of the Rat. *Front Neuroanat* 2**,** 4. doi: 10.3389/neuro.05.004.2008.

Jenkins, A., Franks, Nicholas P., and Lieb, William R. (1999). Effects of Temperature and Volatile Anesthetics on GABAAReceptors *Anesthesiology* 90(2)**,** 484-491. doi: 10.1097/00000542-199902000-00024.

Landisman, C.E., and Connors, B.W. (2007). VPM and PoM nuclei of the rat somatosensory thalamus: intrinsic neuronal properties and corticothalamic feedback. *Cereb Cortex* 17(12)**,** 2853-2865. doi: 10.1093/cercor/bhm025.

Sebel, L.E., Richardson, J.E., Singh, S.P., Bell, S.V., and Jenkins, A. (2006). Additive effects of sevoflurane and propofol on gamma-aminobutyric acid receptor function. *Anesthesiology* 104(6)**,** 1176-1183. doi: 10.1097/00000542-200606000-00012.

Yang, Y., Ou, M., Liu, J., Zhao, W., Zhuoma, L., Liang, Y., et al. (2020). Volatile Anesthetics Activate a Leak Sodium Conductance in Retrotrapezoid Nucleus Neurons to Maintain Breathing during Anesthesia in Mice. *Anesthesiology* 133(4)**,** 824-838. doi: 10.1097/aln.0000000000003493.
